# Embolotherapeutic Strategies for Hepatocellular Carcinoma: 2020 Update

**DOI:** 10.3390/cancers12040791

**Published:** 2020-03-26

**Authors:** Sirish A. Kishore, Raazi Bajwa, David C. Madoff

**Affiliations:** 1 Department of Radiology, Division of Interventional Radiology, Memorial Sloan-Kettering Cancer Center, New York, NY 10065, USA; kishores@mskcc.org (S.A.K.); bajwar@mskcc.org (R.B.); 2 Department of Radiology and Biomedical Imaging, Section of Interventional Radiology, Yale School of Medicine, New Haven, CT 06520, USA

**Keywords:** liver cancer, hepatocellular carcinoma, HCC, embolization, liver-directed therapy, transarterial

## Abstract

Hepatocellular carcinoma (HCC) represents a significant contributor to cancer-related morbidity and mortality with increasing incidence in both developing and developed countries. Embolotherapy as a locoregional therapeutic strategy consists of trans-arterial or “bland” embolization (TAE), trans-arterial chemoembolization (TACE), and selective internal radiotherapy (SIRT). Trans-catheter arterial therapies can be applied along all stages of HCC, either as an alternative or neoadjuvant to surgical resection/transplantation in very early and early stage HCC or as a palliative option for local disease control in unresectable and advanced stage HCC. In advanced stage HCC, SIRT did not demonstrate superiority in comparison to systemic treatment options in several recent large prospective trials, though for carefully selected patients, may confer improved tolerability with similar disease control rates. The latest embolotherapeutic techniques and literature as they pertain to the management of HCC, as well as future directions, are reviewed in this article.

## 1. Introduction

Primary liver cancer, of which hepatocellular carcinoma comprises eighty percent, is the sixth most common cancer worldwide and the fourth most common cause of cancer death [1]. In the United States alone, the incidence of hepatocellular carcinoma (HCC) has almost tripled, becoming the fastest-rising cause of cancer-related deaths [2]. It contributes to 20,578,000 disability life-adjusted years, second only to tracheal, bronchial, and lung cancers [1,3]. 

Locoregional therapies play an important role in patients at all stages of HCC. Transcatheter arterial therapies, or embolotherapy, can serve as an adjunct or alternative to surgical intervention or thermal ablation, in very early and early stage disease, or provide a means of local disease control in patients with intermediate and advanced disease. The objective of this review is to explain the concepts behind the various embolic techniques and summarize the most current available literature reporting the indications, safety, and efficacy of embolotherapy in the management of HCC.

## 2. Embolization Techniques

Approximately 75% of the blood supply to normal liver parenchyma is supplied by the portal vein and the remaining 25% by the hepatic artery. Conversely, due to abnormal neoangiogenesis during carcinogenesis, hepatic malignancy preferentially derives its blood supply from the hepatic artery. For example, a hepatoma that grows to 2 cm is essentially “arterialized” and is supplied almost exclusively by the hepatic artery [4]. This pathophysiologic phenomenon is the mechanistic basis for embolotherapy as a locoregional therapy in the management of HCC [4].

### 2.1. Technical Overview

Transcatheter arterial embolization is an interventional oncology technique whereby a catheter is directed to the artery supplying a tumor, typically via either common femoral or left radial artery. The tumor’s arterial blood supply is then interrupted by the delivery of any of a variety of embolic agents, described below, which defines the specific embolotherapeutic modality. Therapeutic agents are permissively targeted to the tumor based on the differential arterialization compared to normal liver. Pathologic complete response, defined as no viable HCC cells in any of the tumor nodules, is the ideal therapeutic goal underlying all embolization techniques, though sufficient local control can be obtained without complete necrosis depending on the clinical indication for therapy. That being said, the degree of pathological necrosis is a predictor factor of recurrence-free survival and overall survival in post resection and transplant patients [5,6]. 

**Figure 1:**
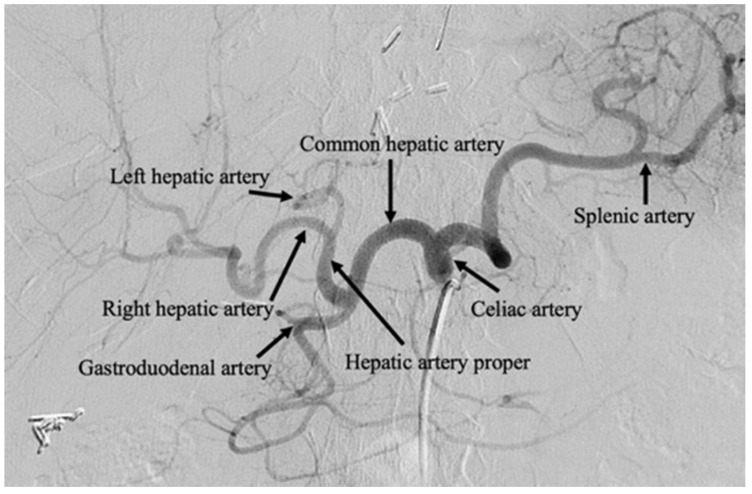
Celiac artery angiogram. Celiac artery angiography performed after selective arterial catheterization of the celiac axis. Transarterial therapies are typically performed beyond the left and right hepatic arteries, with microcatheters positioned in the segmental and subsegmental branches.

Successive generations of therapies have evolved from this principle by also delivering chemotherapeutic, known as transarterial chemoembolization (TACE), and radiotherapeutic, known as selective internal radiation therapy (SIRT), agents. From a clinical perspective, transcatheter arterial embolization therapies can be performed as an outpatient procedure, under moderate conscious sedation or general anesthesia, reserving overnight admission in cases of extensive comorbidity, complication, or for management of substantial periprocedural symptomatology [7,8]. Embolization may be repeated multiple times for local tumor control. Figure 1 contains a celiac artery angiogram delineating the relevant visceral artery anatomy for hepatic embolotherapy.

### 2.2. Transarterial Embolization (TAE)

**Figure 2:**
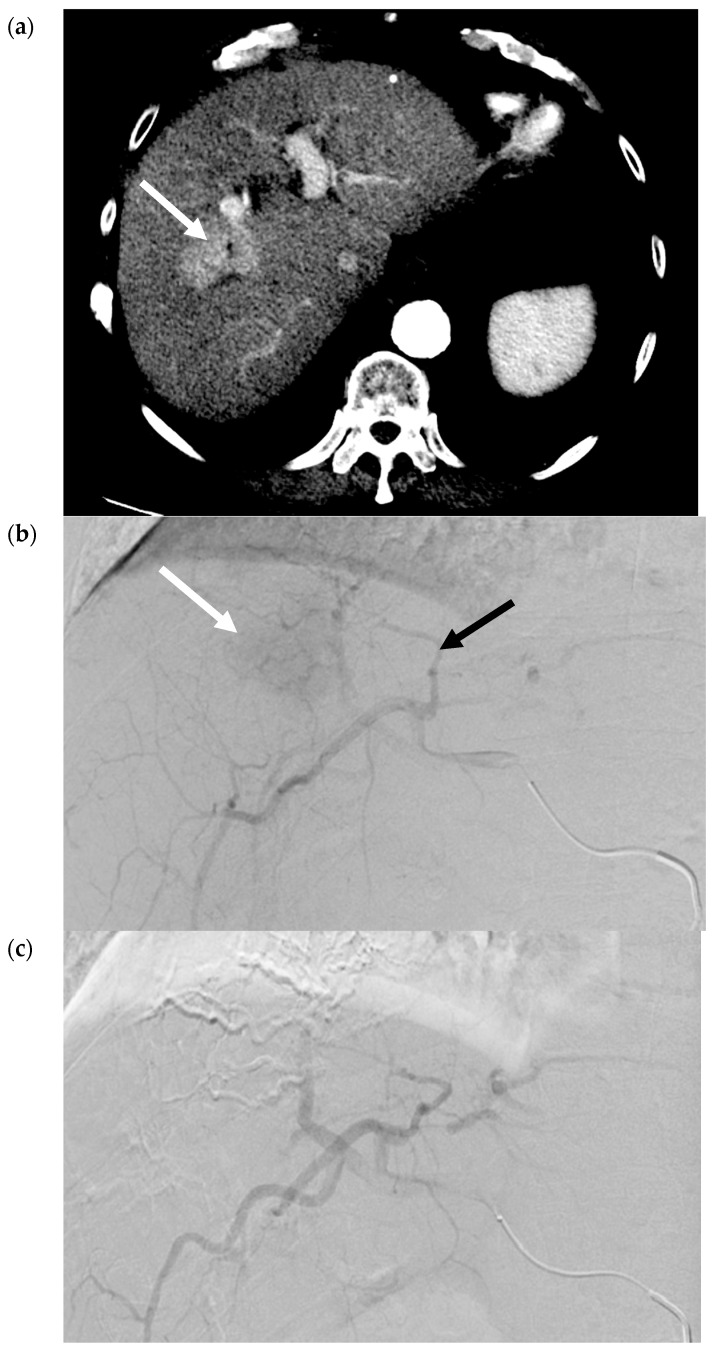

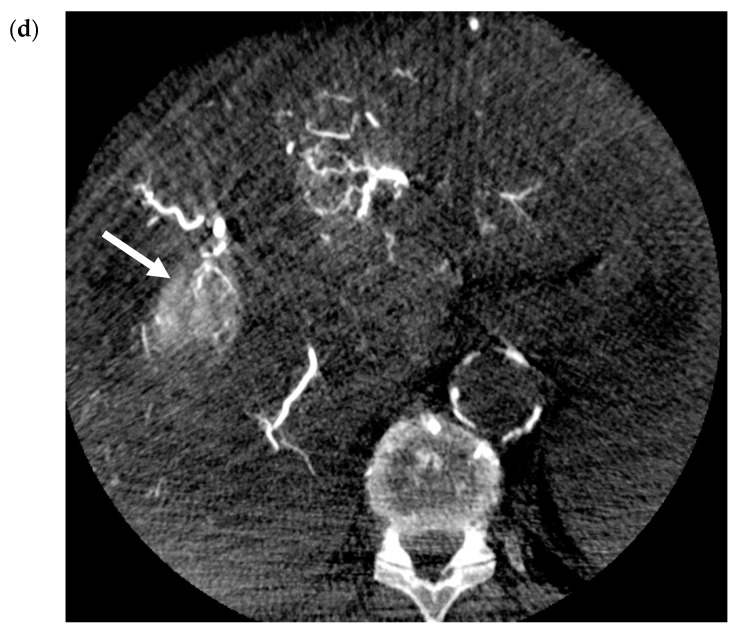
Transarterial Embolization (TAE) of HCC. (**a**) Pre-procedure triphasic contrast-enhanced CT in the arterial phase. Mass with arterial hyperenhancement in segment 8 of the right lobe of liver (white arrow), consistent with hepatocellular carcinoma (HCC). (**b**) Digital subtraction angiogram of the right hepatic artery pre-bland embolization. Mass (white arrow) is supplied by the segment 8 branch of the right hepatic artery (black arrow). (**c**) Digital subtraction angiogram of the right hepatic artery post-bland embolization of the segment 8 branch of the right hepatic artery. A combination of 40-120-µm microspheres and 100-µm polyvinyl alcohol (PVA) particles were used. Angiogram demonstrates the stasis of iodinated contrast in the segment 8 arterial branch with pruning of the distal branches and no opacification of the mass. (**d**) Intra-procedural cone-beam CT immediately post-bland embolization. Retention of iodinated contrast and particles in the treated tumor (white arrow) identical to pre-procedure post-contrast images indicative of complete tumor coverage.

In the context of treating an HCC tumor, polyvinyl alcohol (PVA) particles or gelatin-based microspheres are used most commonly, although alcohol with ethiodized oil and gelatin sponge, have also been described [9]. Arterial deprivation results in an ischemic/hypoxic environment and, consequently, coagulative necrosis in the tumor. The embolic agent can also potentially incite a localized inflammatory reaction and focal angionecrosis [10]. TAE is also known as bland embolization, as the delivered particles do not contain a chemotherapeutic or radioactive agent. Figure 2a–d contain selected images from a bland embolization procedure. The therapeutic endpoint is the stasis of flow in the arteries supplying the tumor with pruning of the distal branches of the treated artery. Completion cone-beam CT should demonstrate the contrast retention within the entire tumor.

### 2.3. Transarterial Chemoembolization (TACE)

**Figure 3:**
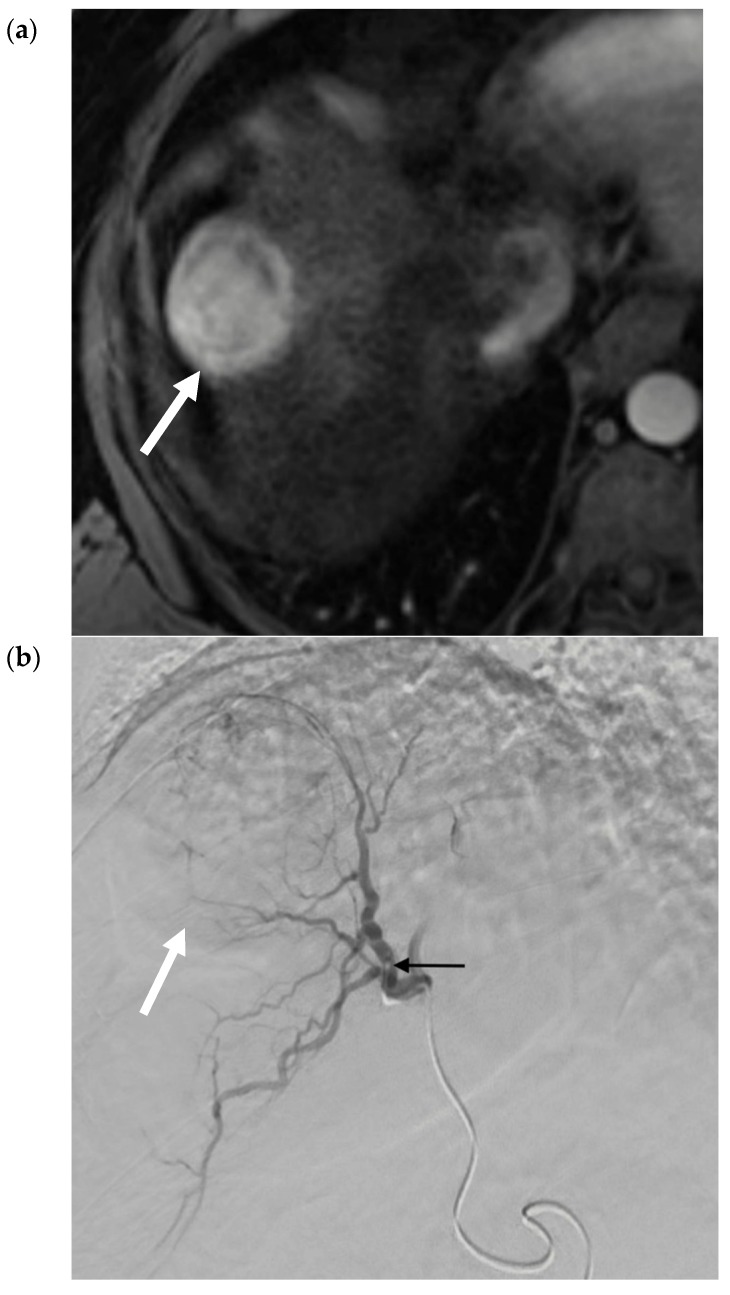

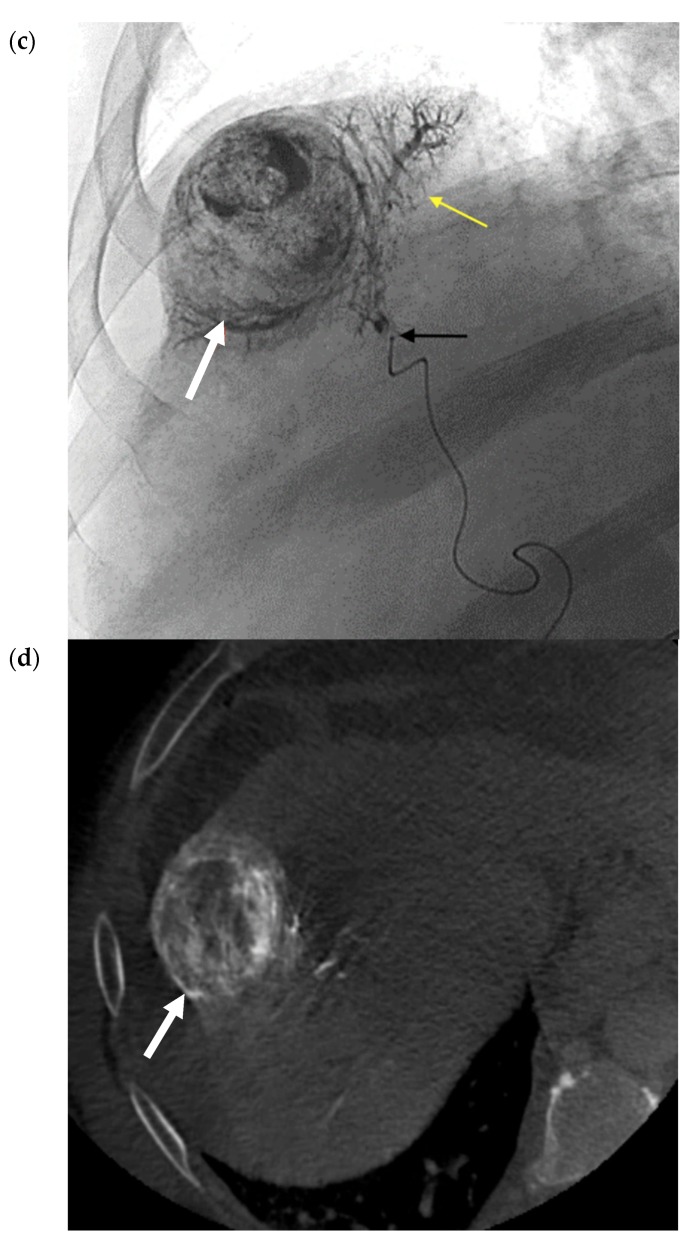
Conventional TACE (cTACE) for Solitary HCC. (**a**) Pre-procedure multiphasic MRI with contrast in the hepatic arterial phase. Hyper-enhancing mass (white arrow) in hepatic segment 8, consistent with HCC. (**b**). Digital subtraction angiogram of the right hepatic artery pre-embolization. Mass (white arrow) is supplied by the segment 8 branch of the right hepatic artery (black arrow). (**c**) Static fluoroscopic image of the treated segment 8 mass post-chemoembolization. Complete embolization of the mass (white arrow) with a diffuse retention of lipiodol after embolization via the right hepatic artery (black arrow). There is lipiodol staining of the portal vein, which is a therapeutic endpoint in conventional trans-arterial chemoembolization (TACE) (yellow arrow). (**d**) Intra-procedural noncontrast cone-beam CT immediately post-chemoembolization. Diffuse circumferential retention of lipiodol in the treated tumor confirms complete tumor treatment (red arrow).

Transarterial chemoembolization delivers chemotherapeutic agents in addition to bland embolic agents to the liver tumor through its predominant blood supply. In conventional TACE (cTACE), an intra-arterial infusion of an emulsion, composed of a chemotherapy agent and lipiodol, a viscous ethiodized oil, is delivered selectively through the catheter. The exact chemotherapeutic agents can be variable but most commonly contain doxorubicin or, in some centers, cisplatin [11]. Next, bland embolization is performed, usually with Gelfoam® (another gelatin-based agent), to occlude the tumor feeding arteries and further sequester the chemoembolic emulsion within the treated tumor [12]. Figure 3a–d contain selected images from a conventional TACE procedure using lipiodol and doxorubicin. Like bland embolization, the therapeutic endpoint should reflect the stasis of flow in the treated vessel, though portal venous “staining” with administered lipiodol is characteristic. The intra-tumoral chemoembolic retention pattern resembles the pre-procedure post-contrast images indicative of complete tumor coverage. The advantages of TACE over systemic chemotherapy include the ability to deliver higher doses of cytotoxic chemotherapy while attempting to minimize the systemic effects.

Despite the best measures being taken, a considerable concentration of the infused chemotherapy agent still enters the systemic circulation [13]. This has led to the development of microspheres loaded with antitumor drugs, most commonly doxorubicin, allowing for a sustained elution of the chemotherapeutic agent, a technique known as drug-eluting bead transarterial chemoembolization (DEB-TACE). There is an immediate embolic effect as a result of the microspheres with sustained elution of the loaded therapy, as systemic concentrations are reduced due to high affinity of drugs to the microsphere carrier [14]. This leads to a decreased peak drug concentration in the systemic circulation. Reduced systemic concentrations may facilitate increased dose delivery and repeated treatments, particularly in patients with other comorbidities [13,15].

### 2.4. Selective Internal Radiation Therapy (SIRT)

Selective internal radiation therapy (SIRT) is a form of brachytherapy whereby microspheres containing the radioactive element yttrium-90 (^90^Y) are delivered transarterially to the liver tumor. SIRT is fundamentally different from TAE and TACE, as the primary mechanism of injury is radiation-mediated via the generation of oxygen free radicals. ^90^Y is an unstable element and undergoes beta decay with a half-life of approximately 64.2 hours. Beta particles have a mean and maximum tissue penetration of 2.5 mm and 10 mm, respectively. SIRT is approved for radiation doses of up to 150 Gy to the target liver, though using advanced techniques such as radiation segmentectomy can result in a dose-to-tumor over 1000 Gy. As a frame of reference, the median dose of stereotactic body radiation therapy for HCC is 54 Gy [16,17]. 

SIRT agents are delivered by way of microspheres in either of two forms: resin-based SIR-sphere® (Sirtex Medical, Sydney, New South Wales, Australia), or glass-based Therasphere® (BTG Corporation, Bothell, WA, USA) [18,19]. Currently, only Therasphere® is FDA approved under a humanitarian device exemption (HDE) as a radiation treatment or neoadjuvant to surgery or transplantation in patients with advanced or unresectable HCC (47). SIR-sphere ® is FDA approved for the treatment of unresectable liver metastases from colorectal cancer. 

Technical advances in radiation dosimetry and catheter technology have led to the development of radiation lobectomy and radiation segmentectomy techniques. Dosimetry calculations are beyond the scope of this article, and readers are directed elsewhere if interested [17,20]. Newer concepts in personalized dosimetry are discussed later in this issue. Briefly, the goal of radiation lobectomy is to provide local tumor control while allowing for potential contralateral hypertrophy. Atrophy and scarring of the treated lobe leads to a slow diversion of the portal vein flow to the untreated lobe and, thus, contralateral hypertrophy in the untreated lobe. Preliminary data in this area demonstrated volumetric changes comparable to portal vein embolization (PVE), albeit slightly slower, with the additional benefit of local tumor control [21].

**Figure 4:**
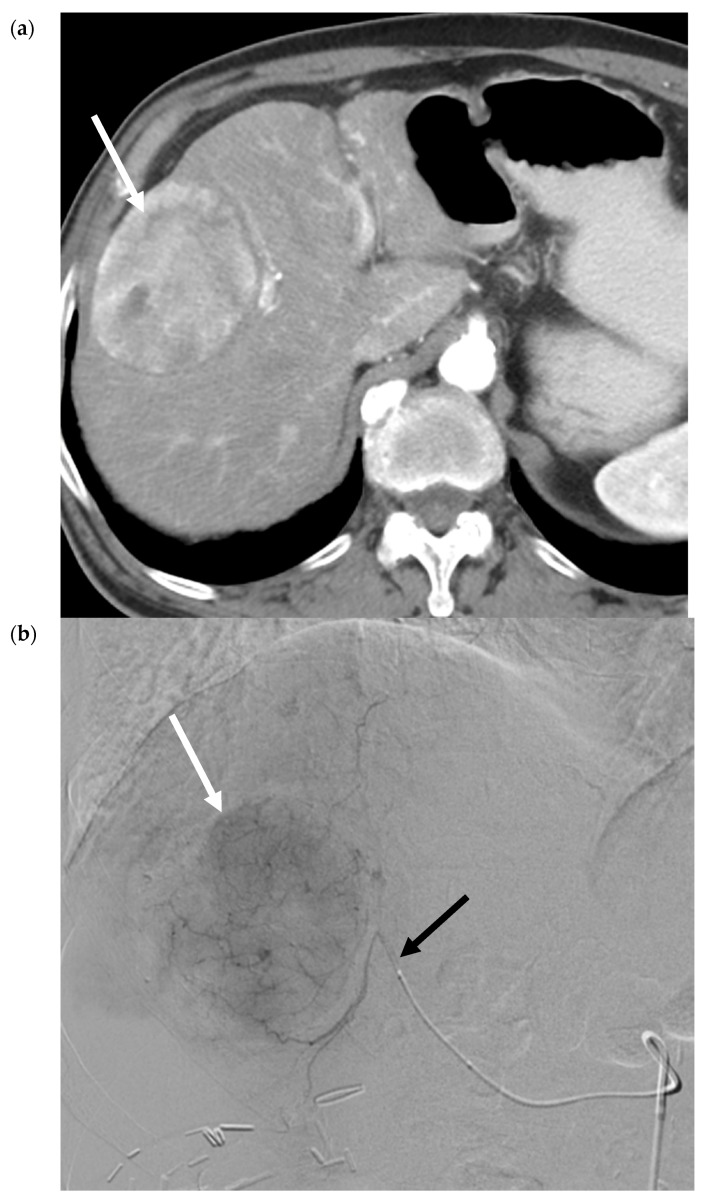

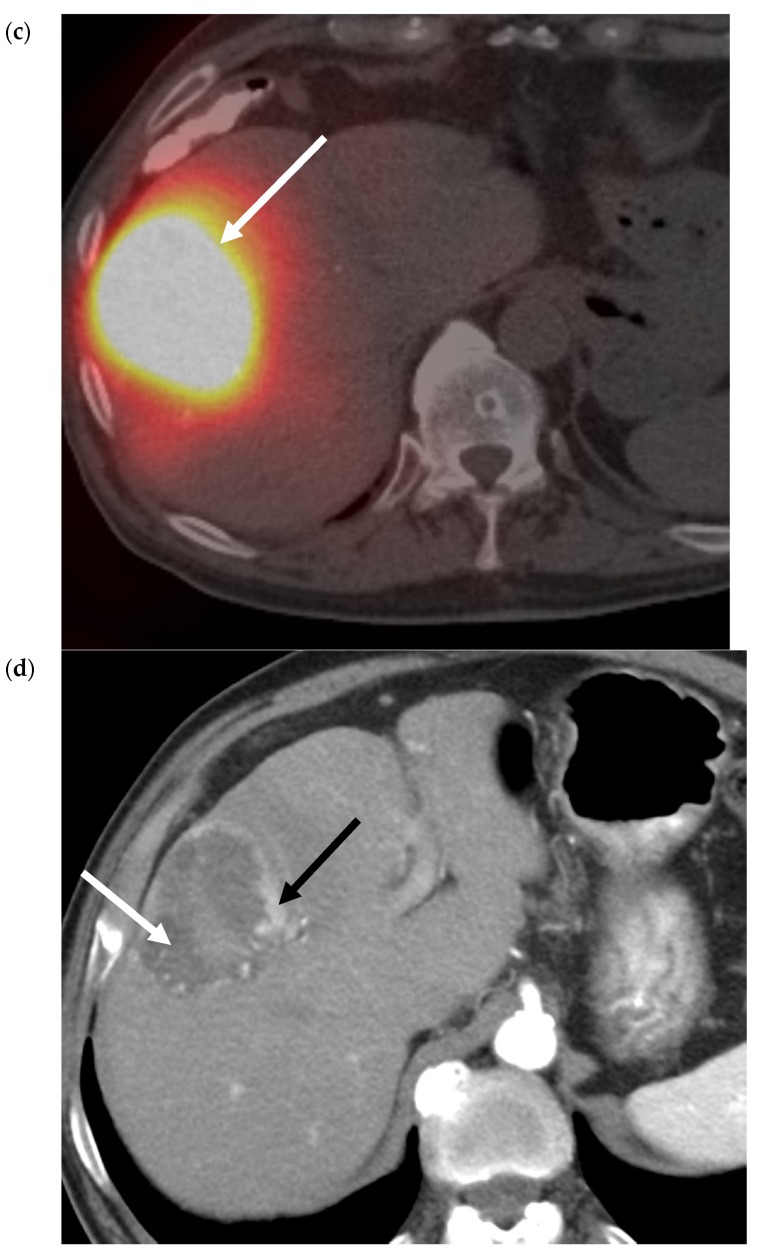
90Y Radiation Segmentectomy for Solitary HCC. (**a**) Pre-procedure triphasic contrast-enhanced CT in the hepatic arterial phase. Mass (white arrow) with arterial hyperenhancement in the anterior sector of the right lobe of the liver, consistent with solitary HCC. (**b**). Digital subtraction angiogram of the anterior division of the right hepatic artery prior to administration of glass 90Y microspheres. Mass (white arrow) is supplied by the anterior division of the right hepatic artery (black arrow). (**c**) SPECT image immediately post-90Y radioembolization. High activity of 90Y microspheres in the liver tumor (white arrow) is demonstrated without evidence of nontarget delivery. (**d**) Post-procedure triphasic contrast-enhanced CT in the arterial phase performed 6 weeks post-treatment. Marked decrease in arterial hyperenhancement of the anterior right hepatic tumor. Overall, the mass is hypoenhancing (white arrow) due to the treatment effect, with a rim and few areas of residual enhancement (black arrow).

Radiation segmentectomy treats ≤2 hepatic segments using a lobar dose, with the goal of “curative” or ablative therapy, by permitting even higher doses of radiation to the liver tumor and minimizing the risk of nontarget embolization [22]. For example, recorded median doses to the tumor range from 536–1200 Gy, whilst limiting the median dose to the treated segment to 210–254 Gy [22,23]. This has been correlated with efficacious tumor necrosis [22,23,24,25]. Growing data and experience has led to the adoption of SIRT as the first-line transarterial locoregional treatment at some centers and suggests further validation as a viable treatment in localized early stage or very early stage HCC, not amenable to percutaneous ablation [26,27]. Figure 4 contains selected images from a 90Y radiation segmentectomy using glass microspheres. Figure 4c confirms delivery of the therapeutic agent to the desired location. Figure 4d demonstrates the gradual involution of tumor, which may not be immediately evident on early post-procedure imaging, as there are areas of residual enhancement. 

## 3. Patient Selection and Applications of Embolotherapy within the Barcelona Clinic Liver Cancer (BCLC) Staging System 

The first step in patient evaluation includes a detailed history and physical exam, as well as laboratory and imaging tests to assess generalized performance status, hepatic function, tumor burden, and vascular anatomy. Patients with an incompetent or recently cannulated Sphincter of Oddi are often given pre-procedure and post-procedure antibiotics. Infectious complications (cholangitis and liver abscess) and bilomas are related to previous biliary tree intervention [28,29]. Prior intervention, such as endoscopic retrograde pancreatography and/or stenting, leads to an incompetent Sphincter of Oddi and, thus, bacterial colonization of the biliary tree. Necrotic tumor then provides an ideal growth medium for bacteria. Abscess formation in those with an incompetent sphincters range from 0–15%, compared to 1–2% in patients with a functional sphincter [30].

While controversial, many standard contraindications are considered relative depending on the operator, severity of comorbidity, treatment extent, and overall clinical scenario. General contraindications to transcatheter arterial therapies can be categorized as follows [31,32,33]:

1. Patient factors
  - Performance status (Eastern Cooperative Oncology Group ≥ 2)
  - Renal failure (creatinine ≥2.0 mg/dL)
  - Unfavorable anatomy (inability to prevent arteriovenous shunting or nontarget embolization)
2. Liver factors
  - Decompensated liver disease (Child-Pugh B ≥ 8)
  - Alterations to portal vein flow (transjugular intrahepatic portosystemic shunt, thrombosis, and hepatofugal flow)
3. Tumor factors
  - Extensive tumor with complete replacement of both lobes

HCC staging is complex, and the Barcelona Clinic Liver Cancer (BCLC) Staging and Treatment Strategy is the most commonly employed system [34]. Within the BCLC system, three domains are evaluated: tumor extent, liver function/cirrhosis stage, and performance status. Tumor stage is assessed by the number and size of the lesions, as well as vascular invasion and the presence of extrahepatic disease. Liver quality is determined by the status of the underlying parenchyma (cirrhosis), liver function tests stratified by the Child-Pugh score, ICG, portal hypertension, and the future liver remnant (FLR). The Eastern Cooperative Oncology Group (ECOG) scale is used to summarize the performance status [35]. 

### 3.1. Very Early Stage (0) or Early Stage (A)

For a single tumor ≤2 cm (very early stage) or solitary/up to three tumors ≤3 cm (early stage), surgical resection or transplant is recommended, respectively [36,37]. However, due to surgical contraindications in the cirrhotic patient population, as well as limited donor availability, both options are often not available [38,39]. Furthermore, in spite of the curative intent, recurrence rates within five years of surgery remain high [40]. Ablation is recommended in unsuitable surgical candidates or those with strong preferences towards more minimally invasive options [37,41]. Transcatheter embolotherapies also have a role in this cohort of patients, including acting as an adjunct to surgical resection, a bridge or downstaging tool to facilitate transplantation, or as a primary treatment in patients who are deemed unsuitable surgical candidates. 

#### 3.1.1. Adjunct to Surgery

Currently, patients with resectable tumors but an inadequate future liver remnant (FLR), portal vein embolization (PVE) is advocated to induce contralateral liver hypertrophy. Factors to be considered when evaluating patients for PVE include the degree of underlying liver disease and the extent of the planned surgery. Generally speaking, for patients with normal hepatic function, a predicted FLR of ≤ 20% is an indication for PVE; however, in patients with underlying cirrhosis, a FLR ≤ 40% is the recommended threshold. Strict patient selection is important to limit the risk of hepatic decompensation from PVE, such as patients with advanced cirrhosis who may not only experience an increased risk of hepatic decompensation from PVE but may also sustain limited FLR hypertrophy after PVE [42]. PVE is reported to induce a FLR hypertrophy of 10–46% after 2–8 weeks [43]. However, during this window, there is an increased risk of tumor progression, which can preclude curative surgical resection [44]. Consequently, transarterial techniques may provide an important adjunct to surgery in combination with PVE or as a standalone technique, offering both local tumor control as well as FLR hypertrophy. Specifically, TACE combined with PVE, or radiation lobectomy alone, have been shown to induce FLR hypertrophy in an anticipation of hepatic resection [21,45,46].

In patients with large HCC (>5 cm), a systematic review and meta-analysis reported a higher rate of surgical resection in sequential TACE and PVE than PVE alone (90% versus 75%; *p* < 0.001) [46]. Additionally, sequential TACE and PVE can provide additional local tumor control over PVE alone with a complete pathologic necrosis of 83% versus 5.5%; *p* < 0.001 [47]. Finally, in patients undergoing major hepatectomy, cTACE and PVE are associated with better overall survival and recurrence-free survival than PVE alone [48,49]. 

Radiation lobectomy has demonstrated volumetric changes comparable to PVE, albeit slightly slower, but with the additional benefit of local tumor control (when PVE is not combined with TACE). A systematic review, which included 215 patients with HCC (out of 312 included), found rates of contralateral liver lobe hypertrophy following radioembolization ranged from 26% to 47% at 44 days–9 months. One of the included studies compared SIRT directly to PVE, reporting significantly greater hypertrophy in the PVE group, 61.5% versus 29.0% (*p* < 0.001) within a shorter median time frame, 33 days (range 24–56 days) (SIRT: 46 days (range 27–79 days)) [50]. The exact kinetics of FLR hypertrophy in SIRT remain elusive, possibly related to underlying patient and disease characteristics, as well as potential differences in mechanisms and rates of hypertrophy from radiation versus immediate portal vein occlusion. Surgery following radiation lobectomy is reported to be feasible and safe, offering curative surgery to a cohort of patients initially staged as unresectable while providing local tumor control [51].

#### 3.1.2. Bridge to Transplant

Orthotopic liver transplant is advocated in stage A patients falling within the Milan Criteria or University of California, San Francisco criteria. Currently, dropout rates range from 8.9–9.4% at six months and up to 19.6% at 12 months [52,53]. Furthermore, in the United States, the United Network for Organ Sharing (UNOS) has introduced a six-month delay in the assignment of exception points for patients with HCC. This is to enable more equitable organ donation and gain insight into the tumor biology of the transplant candidate to optimize long-term outcomes [54]. UNOS has assigned automatic priority to downstaged patients owing to low recurrence rates and excellent post-transplant survival. Similarly, patients with an alpha-fetoprotein (AFP) < 500 following locoregional therapy were assigned automatic priority [55]. 

Transcatheter arterial techniques can either be used as a bridge to therapy by reducing the alpha-fetoprotein (AFP), reducing drop-off/mortality for candidates on the waitlist, or downstaging tumors to within the T2 category. In patients with HCC within the Milan criteria, bridging therapy is estimated to reduce the dropout rate to 0–10% [56]. Bland, chemotherapeutic, and radiotherapeutic embolization techniques have all shown to be similar bridging therapies in terms of safety and efficacy [57,58,59]. A recently conducted prospective study comparing ^90^Y to cTACE in patients with either BCLC stage A or B found longer times to progression: >26 months in the ^90^Y group versus 6.8 months in the cTACE group, *p* = 0.012, (HR 0.122, 95% CI, 0.027–0.557, *p* = 0.007), but similar tumor necrosis and median survival times. The authors concluded radioembolization provides longer tumor control and could reduce dropout from transplant waitlists [60].

Patient responses to locoregional techniques may provide important insight into their tumor biology; complete pathologic response on explant reduces HCC recurrence and improves post-transplant survival [6]. Thus, locoregional therapy can benefit candidates with tumors within the UNOS T2 category or who meet the Milan criteria with wait times greater than six months. Furthermore, those candidates with an ineffective response or interval progression can be removed from the waiting list based on anticipated long-term prognosis [61].

#### 3.1.3. Downstage to Transplant

The American Association for the Study of Liver Diseases suggests patients beyond the Milan criteria (T3) should be considered for liver transplantation if successfully downstaged into the Milan criteria [37]. Traditionally, TACE has been the most widely used bridging/downstaging therapy; however, with the increasing use of radioembolization, the optimal locoregional therapy remains undetermined [37]. A retrospective study reported a higher downstaging rate for UNOS T3 category tumors treated by radioembolization compared to TACE, 58% versus 31% (*p* = 0.023), with a trend towards a longer time to progression, 33.3 months versus 18.2 months (*p* = 0.098) [62]. 

A systematic review and meta-analysis reported a 0.48% (95% CI, 0.39–0.58%) pooled success rate of downstaging HCC to within the Milan criteria, with no difference between TACE and radioembolization for successful downstaging (*p* = 0.51) [63]. There was no difference between either modality for recurrence. The recurrence rate after transplant did not differ significantly by the downstaging modality (0.17 for TACE versus 0.26 for radioembolization; *p* = 0.40); however, results were imprecise, with significant heterogeneity between studies. Survival data were not possible due to the study heterogeneity. Toso et al. noted a higher rate of post-transplant tumor recurrence in patients who underwent downstaging therapy to tumor parameters within the Milan criteria, 11%, versus those patients always within the criteria, 1.7% (*p* = 0.001). However, no difference in the five-year disease-free survival between these groups was noted in this retrospective study [64].

#### 3.1.4. Unsuitable Surgical Candidates

Ablation is recommended in the guidelines for patients with unresectable tumor(s) or contraindications to surgical resection or transplantation in this category [37,41]. It is typically employed in patients with tumors <3 cm and no vascular invasion [65]. Other contraindications include locations near critical structures, such as <1 cm to a main biliary duct, diaphragm, or heart or treatment in a patient with a dilated biliary system [66]. 

While it may be difficult to achieve complete ablation of larger tumors, combined embolization-ablation may provide a valuable treatment option for tumors beyond the size threshold. A systematic review and meta-analysis reported the overall survival rate was superior in patients with intermediate-size tumors, defined as 3–5 cm (at one year OR = 3.46, 95% CI: 1.29–9.28, *p* = 0.01; three years OR = 3.58, 95% CI: 1.79–7.15, *p* = 0.0003; and five years OR = 5.34, 95% CI: 2.42–11.75, *p* < 0.001), and large-size tumors, defined as > 5 cm (at one year OR = 2.91, 95% CI: 1.60–5.29, *p* = 0.0004 and three years OR = 6.69, 95% CI: 3.01–16.07, *p* < 0.001), who underwent radiofrequency ablation (RFA) plus TACE than in those who underwent RFA monotherapy [67]. In a retrospective series of patients who underwent treatment of solitary tumors up to 7 cm by either surgical resection or combined embolization-ablation, long-term follow-up of over ten years showed no difference in intrahepatic or metastatic progression or overall survival [68]. Patients in the surgical group experienced more complications (*p* = 0.004), longer hospitalizations (*p* < 0.001), and an increased likelihood of hospital readmission within 30 days of discharge (*p* = 0.03) compared to the embolization-ablation group [68].

Conversely, ablation can be contraindicated, often due to anatomic considerations, thereby requiring an alternative locoregional therapeutic option. A retrospective study examining differences in five-year overall survival for patients with a single-nodule HCC of 3 cm or smaller, without vascular invasion, found no differences in OS between resection, ablation, and TACE [69]. Kim et al. studied TACE versus RFA for the treatment of a single HCC ≤ 2 cm and found that RFA yielded a longer time to progression but no difference in overall survival at five years [70]. 

Radiation segmentectomy can also potentially be used as an alternative to percutaneous ablation, particularly in patients with unfavorable anatomic locations. When assessing the efficacy of radiation segmentectomy for 102 patients with tumors deemed to be “unablatable”, Vouche et al. reported that the median time to disease progression was 33.1 months, and the median overall survival was 53.4 months (34.5 months when censored to transplant) [71]. Thirty-three of those patients were transplanted with a median time to transplantation of 6.3 months (3.6–9.7 months). Explant pathology revealed a complete pathologic response in 17 (52%) and a partial response in 16 (48%) of patients.

A retrospective propensity score matched study compared the efficacy of radiation segmentectomy and segmental TACE in the patients with unresectable, solitary HCC ≤ 3 cm [72]. Radiation segmentectomy led to greater target and overall responses (94.7% and 92.1% compared to 47.4% and 52.6%; *p* = 0.004 and *p* = 0.005, respectively) and longer time to secondary therapy (median 812 (363–812) versus 161 months (76–350); *p* = 0.001). There was no difference in toxicity or overall survival. A disadvantage of radioembolization is the much higher cost compared to TACE [73]. 

### 3.2. Intermediate Stage (B)

BCLC intermediate stage comprises multinodular HCC greater than 3 in number and/or lesions > 3 cm with relatively preserved liver functions (CP score < 9) and performance statuses (ECOG 0). The goals of therapy in this heterogenous stage range from downstaging to curative resection/transplant to palliative treatment to delay progression and improve overall survival. Initial work in this area by Llovet et al. demonstrated that survival probabilities at one year and two years were 82% and 63% for TACE and 63% and 27% for control (*p* = 0.009). Embolic therapy remains a balance between the maximal tumor response and minimal hepatocellular toxicity. Currently, the only recommended therapy in the BCLC staging system for downstaging is TACE. A phase II clinical trial explored the potential improvement in the efficacy and safety of the TACE-sorafenib combination compared to TACE alone in intermediate-stage HCC. The trial found the median time to progression was similar in both groups, 169 (TACE-sorafenib) versus 166 days (TACE-placebo) (HR: 0.797, *p* = 0.072) [74]. Similarly, no difference in overall survival was recorded (HR 0.898, 95% CI, 0.606–1.330, *p* = 0.295), with the median overall survival not reached in either group. The most commonly reported adverse events with all-grade differences >10% across the arms (sorafenib versus placebo) included diarrhea (52.9% versus 17.2%), hand-foot skin reaction (46.4% versus 6.6%), anorexia (30.7% versus 20.5%), hypertension (30.1% versus 16.6%), hepatobiliary (23.5% versus 11.3%), rash (21.6% versus 7.3%), and weight loss (20.3% versus 1.6%). No unexpected adverse events related to sorafenib were observed. Four deaths were recorded in the TACE-sorafenib group, compared to one in the TACE-placebo group. Radioembolization is increasingly being used for downstaging, as described earlier. 

Also, radioembolization is more commonly being employed in the treatment of unresectable HCC. A meta-analysis of three randomized controlled trials, including the SIRTACE and PREMIERE trials, found no differences in terms of progression free or overall survival between TACE and SIRT. Of note, however, the PREMIERE trial was one of the first randomized controlled trials to compare SIRT to TACE in BCLC stage A/B patients and demonstrated a longer time to progression, 14.5 months (SIRT) versus 6.4 months (TACE) (*p* = 0.0019) without significant difference in overall survival (23.8 months versus 17.7 months, *p* = 0.9772) [75]. On evaluation of the three trials, there were significant study limitations, such as unmet predetermined sample sizes and inconsistent and imprecise results, probably due to differences in the study populations, BCLC stage, and reported outcomes [76]. However, in a study examining patients’ refractory to TACE, 36.7% patients underwent SIRT and demonstrated a response on imaging, with 10% successfully downstaged and receiving a liver transplantation [77]. 

### 3.3. Advanced Stage (C)

Sorafenib and lenvatinib are recommended first-line therapies for advanced HCC, followed by nivolumab as the second line [78]. Sorafenib confers a modest survival increase at the expense of considerable drug-related toxicity, most commonly, diarrhea, weight loss, hand–foot skin reaction, and hypophosphatemia [79]. Locoregional therapy in the form of SIRT and TACE has been applied in this setting to provide local disease control in patients with acceptable hepatic function and disease burden, with fewer systemic side effects than systemic therapy. It was postulated that administering sorafenib in combination with TACE might be useful to counteract TACE-induced angiogenic factors and, therefore, improve outcomes from TACE treatment [80]. Final analysis of the GIDEON study exhibited a trend of longer overall survival in patients whom received a prior TACE or TACE-sorafenib combination compared to sorafenib alone, without safety concerns [81]. A systematic review and meta-analysis showed TACE-sorafenib combination therapy significantly improved the time to progression (HR = 0.66, 95% CI 0.50–0.81, *p* = 0.002) and a trend to improve the overall survival (HR = 0.63, 95% CI 0.55–0.71, *p* = 0.058) compared to TACE alone. Additionally, the disease control rate, defined as a combination of complete response rate, partial response rate, and stable disease rate, increased significantly in the combination therapy group (OR = 2.93, 95% CI 1.59–5.41, *p* = 0.005) [82]. 

SIRT was originally approved as a palliative treatment option in patients with advanced HCC; however, the specific indications for SIRT in advanced HCC remains undefined in BCLC guidelines [83]. The SARAH trial, a phase III trial designed to show superiority comparing SIRT to sorafenib in 467 patients with BCLC stage C, recurrent tumor ineligible for curative surgical therapy or tumors refractory to TACE, Child-Pugh score A–B and ECOG 0–1, did not show a significant difference in OS between the two groups (8.8 months in the SIRT group versus 9.9 months in the sorafenib group (HR 1.15, 0.94–1.41, *p* = 0.18) [84]. A post-hoc analysis of patients with a tumor burden ≤25% and well-preserved liver function (albumin-bilirubin grade 1) in the intention to treat the population showed a nonsignificant trend towards increased OS and PFS in the SIRT group, suggesting a potential niche for SIRT despite the small subgroup sizes [85]. However, this finding needs to be validated in larger studies specifically targeted to this subgroup of advanced HCC patients.

Results similar to the SARAH trial were encountered in the SIRVENIB trial, conducted in the Asia-Pacific region, whereby median OS in intention-to-treat populations were 8.8 and 10.0 months with SIRT and sorafenib, respectively (HR 1.01, 95% CI 0.81–1.25, *p* = 0.9529). It is important to note, as Llovet and Finn have commented, no differences in survival does not mean that SIRT is equivalent to sorafenib [86]. To show noninferiority, new trials must be conducted with predetermined noninferior margins. Sposito and Mazzaferro discuss further drawbacks of both the SARAH and SIRVENIB trials, particularly the possible dilutional effect of the study due to multi-centrality, lack of standardized dosimetry, and median wait time of 21–29 days to receive SIRT compared to 3–7 days to receive sorafenib [87].

The use of SIRT-sorafenib combined therapy in patients with BCLC B and C, not eligible for TACE, was also evaluated in the SORAMIC study [88]. The study failed to demonstrate a survival benefit in the intention to treat the palliative arm of the study for patients who received SIRT and sorafenib combination therapy; median overall survival was 12.1 months compared to sorafenib alone (11.4 months) (HR: 1.01, 95% CI: 0.82–1.25, *p* = 0.93). However, subgroup analyses of the preprotocol population indicated a survival benefit for SIRT and sorafenib combination in patients without cirrhosis (HR 0.46, 0.25–0.86, *p* = 0.02); cirrhosis of nonalcoholic etiology (HR 0.63, *p* = 0.012); or patients ≤65 years old (HR 0.65, *p* = 0.05). Completion of the STOP-HCC trial (NCT01556490) is anticipated in February 2020.

HCC portal vein tumor thrombus, or macrovascular invasion, can be present at diagnosis or develop later in the natural history of the disease. HCC invading the portal vein has a very poor prognosis, with a median survival of 2–4 months [89]. A retrospective study comparing SIRT to sorafenib in patients with HCC and portal invasion found an overall median survival benefit in patients treated with SIRT compared to sorafenib: 26.2 months versus 8.7 months, respectively (HR 0.40, 95% CI: 0.19–0.82) (*p* = 0.013). The difference in overall survival was more pronounced in patients with branch portal vein thrombosis (as opposed to main portal vein thrombosis), with a respective median OS of 25.3 months (95% CI 13.8–36.8) versus 7.0 months (95% CI 5.2–8.9) (*p* = 0.001), than in cases of main PVT, with a respective median OS of 12.0 months (95% CI 4.6–19.3) versus 6.5 months (95% CI 4.8–8.3) (*p* = 0.195) [90]. Furthermore, SIRT has been shown the long-term preservation of health-related quality of life in patients with portal vein invasion [91] 

## 4. Future Therapies

Precision medicine has provided a major breakthrough in cancer care. Genetic mutations and molecular signaling pathways, as documented in the survival benefit of small molecule inhibitors, have been implicated in the carcinogenesis and progression of hepatocellular carcinoma. A number of new small molecule inhibitors, epigenetic regulators, and monoclonal antibodies are being investigated for their safety and efficacy in advanced HCC [92].

One particular area of interest lies in the role of immunotherapy and potential synergy with locoregional therapy. Immune cells and T-cell infiltration correlate with HCC outcome and prognosis, respectively [93]. Nivolumab and pembrolizumab, two immune checkpoint inhibitors, have been FDA approved for second-line systemic therapy in patients with advanced HCC after failing sorafenib [37]. Trials have shown that the immune response elicited after an HCC has been treated by ablation/embolization can be enhanced by PD-1 inhibition [94]. A number of trials are being conducted to evaluate potential synergistic therapeutic effects between anti-PD1 inhibitors and SIRT and TACE [95]. Preliminary studies assessing the safety of SIRT-PD1 inhibitor combination therapy has been shown to be safe [96]. Checkpoint inhibitors are currently approved for unresectable HCC in the USA and China. However, they have shown efficacy in European and Asian cohorts too [97].

De Toni proposes an initial systemic therapy with an immune checkpoint inhibitor and antiangiogenic therapy, reserving TACE for those lesions demonstrating radiological progression [98]. De Toni’s approach is based on an early objective response seen in one-third of patients prescribed checkpoint inhibitors and employing TACE sparingly to limit potential collateral damage to normal hepatic parenchyma. This study design is implemented in the ongoing randomized study DEMAND (NCT04224636), where candidates receive atezolizumab and bevacizumab and are then randomized to receive synchronous or on-demand selective TACE.

Artificial intelligence has the potential to revolutionize all aspects of healthcare, particularly image-guided therapy. For example, incomplete HCC treatment via TACE can result in increased levels of angiogenic factors, leading to tumor growth and metastasis [99]. A novel study conducted by the MD Anderson Cancer Center Group demonstrated that machine-learning may play a role in predicting patient responses to TACE using quantitative imaging features in combination with the BCLC staging system. The group concluded such an approach is likely to provide useful information for aiding HCC patient selection for TACE [100]. As our ability to identify potential responders using computational technology and biological insight improves, the role of embolotherapy in HCC has the potential to expand beyond the confines of traditional classification systems and large trial data that can be limited in the ability to define the optimal role for this therapeutic modality.

## 5. Conclusions

Embolotherapy is an important element in the management of all stages of HCC. The therapeutic agents and indications of transarterial therapies have expanded, proposing options to patients ranging from a curative treatment in early/very early stages to improved overall survival and a well-tolerated palliative treatment in advanced stage/portal vein invasion with improved quality of life over systemic therapy. In particular, with the increasing use and availability of technology, selective internal radiotherapy offers benefits to patients across the BCLC staging spectrum. Nonetheless, many clinical guidelines’ recommendations are hampered by the quality of available literature in spite of the quantity, and future trials should focus on specific and clinically relevant outcomes with more refined patient selection criteria. Additionally, parallel advances in immunotherapy and artificial intelligence offer the promise of new paradigms in the treatment of patients with HCC.
